# Editorial: Neuroimaging in affective neuroscience

**DOI:** 10.3389/fnhum.2026.1945667

**Published:** 2026-08-05

**Authors:** Daniele Corbo, Umer Asgher, Jintao Sun, Lisa Hahn

**Affiliations:** 1 Department of Medical and Surgical Specialties, Radiological Sciences and Public Health, University of Brescia, Brescia, Italy; 2 National Center of Artificial Intelligence (NCAI), National University of Sciences and Technology (NUST), Islamabad, Pakistan; 3 School of Interdisciplinary Engineering and Sciences (SINES), National University of Sciences and Technology (NUST), Islamabad, Pakistan; 4 Department of Neurology, The Affiliated Brain Hospital of Nanjing Medical University, Nanjing, China; 5 Department of Psychiatry and Psychotherapy, University Hospital, Ludwig-Maximilian University, Munich, Germany; 6 German Center for Mental Health, Partner Site Munich, Augsburg, Germany

**Keywords:** affective neuroscience, clinical neuroimaging, emotion regulation, functional connectivity, imaging transcriptomics, interoception, network neuroscience, resting-state fMRI

## Abstract

**Figure:**
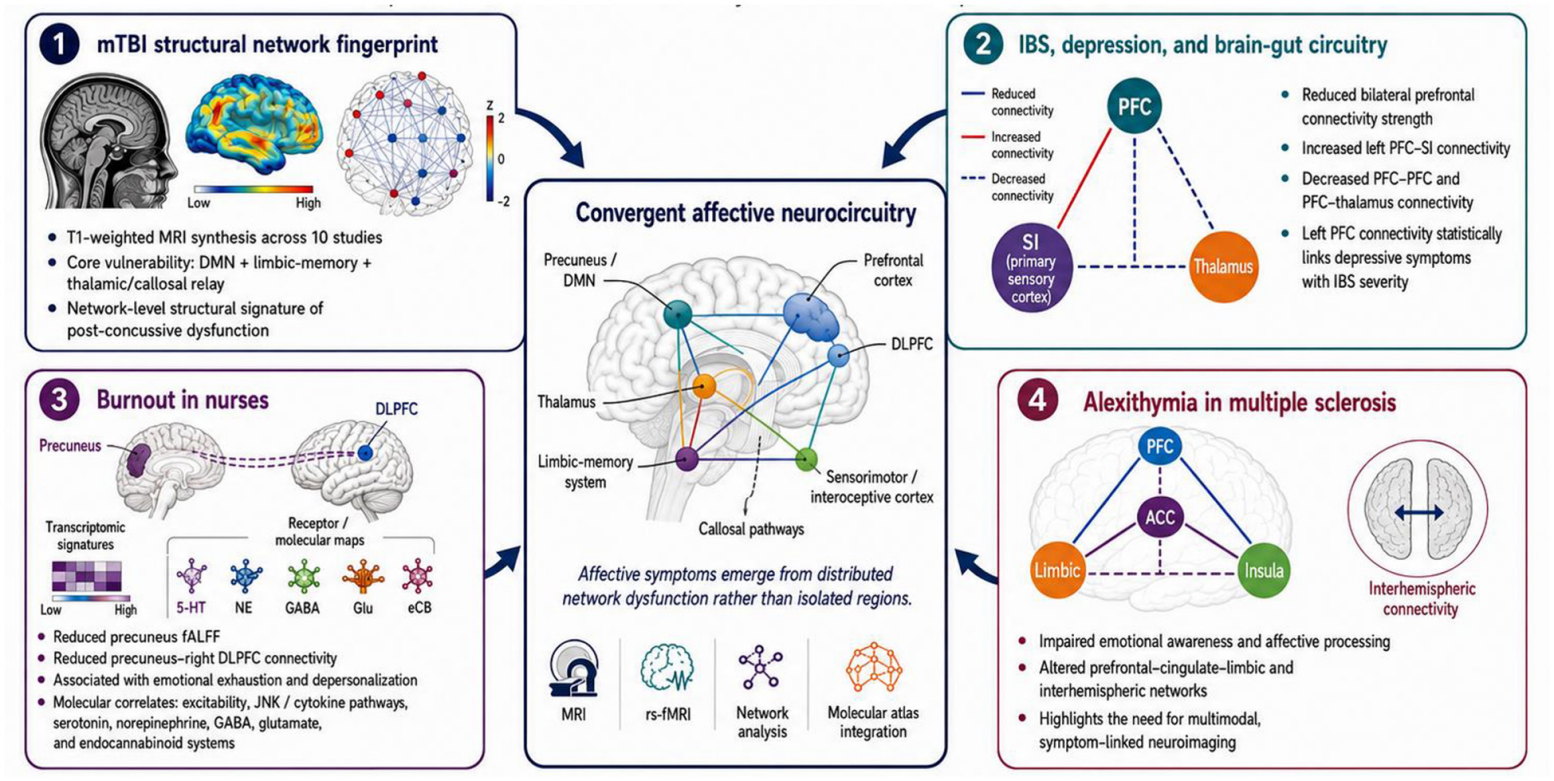
Graphical abstract–Neuroimaging in affective neuroscience.

Graphical abstract–Neuroimaging in affective neuroscience.

## 1 Premise

Affective neuroscience seeks to explain how the brain integrates interoceptive, sensory, mnemonic, motivational, and contextual information to generate affective states and regulate behavior. Neuroimaging has been central to this effort, but the field has progressively moved beyond one-region-one-emotion accounts. Contemporary affective neuroimaging increasingly treats emotion as an emergent property of distributed, dynamic, and context-sensitive systems that span cortical, subcortical, brainstem, and white-matter networks. This shift is accompanied by a parallel methodological transition: from regional activation maps to analyses of intrinsic activity, large-scale functional connectivity, structural network topology, and spatial correspondence with molecular atlases. This transition also demands precision in interpretation. Blood-oxygen-level-dependent signals, morphometric measures, and electrophysiological indices are indirect and model-dependent correlates of neural processes; they do not constitute direct measurements of an emotion or subjective experience. Functional connectivity denotes statistical dependence between regional time series rather than a direct anatomical pathway, while spatial correspondence with transcriptomic or neurotransmitter atlases cannot be equated with participant-specific molecular measurement. The value of affective neuroimaging therefore lies not in reducing emotion to a single biomarker, but in testing mechanistic models that connect distributed brain organization to carefully defined behavioral, clinical, and experiential phenotypes.

The four contributions assembled in this Research Topic reflect this multiscale direction as shown in Graphical Abstract. They comprise two original investigations, one systematic network synthesis, and one mini-review, spanning irritable bowel syndrome (IBS), mild traumatic brain injury (mTBI), occupational burnout, and multiple sclerosis (MS). Although these conditions differ substantially in etiology and clinical expression, the studies collectively emphasize interactions among self-referential processing, interoceptive integration, executive control, and affect regulation. Recurrent involvement of the default mode network (DMN), prefrontal systems, thalamic relay structures, and interhemispheric pathways suggest a transdiagnostic architecture of vulnerability, while also underscoring that similar network nodes can participate in distinct disease mechanisms.

## 2 Affective-interoceptive coupling in irritable bowel syndrome

Liu et al. examined the neural interface between depressive symptoms and gastrointestinal symptom burden using resting-state functional magnetic resonance imaging (rs-fMRI). Their exploration cohort included 32 patients with IBS and 37 healthy controls. The analytical pipeline combined voxel-wise functional connectivity strength (FCS), seed-to-whole-brain connectivity, and AAL atlas-based seed-to-seed connectivity. Relative to controls, patients with IBS showed reduced bilateral prefrontal FCS, increased coupling between the left prefrontal cortex and primary somatosensory cortex, and reduced connectivity between the left and right prefrontal cortices and between the left prefrontal cortex and bilateral thalamus. The left angular gyrus, a DMN hub, also displayed altered connectivity with prefrontal, precentral, somatosensory, and temporal regions.

The most important contribution is the identification of a prefrontal-sensorimotor-thalamic configuration linking affective symptoms to visceral symptom severity. Lower prefrontal FCS was associated with greater depressive and IBS symptom burden, and prefrontal FCS statistically mediated the association between depression scores and IBS severity. This finding is compatible with a model in which impaired top-down prefrontal regulation and altered sensory-interoceptive integration jointly contribute to symptom amplification. However, because the study is cross-sectional, the mediation model should not be interpreted as evidence of temporal or biological causation. Longitudinal studies, task-based paradigms involving visceral stimulation or emotion regulation, and concurrent autonomic, inflammatory, and gastrointestinal measurements will be required to establish directionality and mechanism.

## 3 A structural network fingerprint of mild traumatic brain injury

Mavroudis et al. addressed a different level of analysis by synthesizing T1-weighted morphometric findings across 10 adult mTBI studies. Approximately 35 atlas-defined regions were encoded in a region-by-study matrix, with structural decreases and increases represented directionally. The authors then applied network-level abnormality metrics, co-alteration graph modeling, principal component analysis, and hierarchical clustering. Across heterogeneous cohorts and analytic approaches, a DMN-limbic-thalamic core emerged, with additional involvement of frontoparietal, salience, callosal, sensorimotor, and brainstem-cerebellar systems.

The study is conceptually important because it reframes mTBI as a disorder of distributed structural vulnerability rather than a collection of isolated morphometric abnormalities. Midline DMN regions, the hippocampus, thalamus, cingulate cortex, and prefrontal association areas are directly relevant to memory, self-referential processing, arousal, attention, and affective regulation, thereby providing a system-level context for persistent cognitive and emotional symptoms after apparently mild injury. At the same time, the structural network fingerprint is derived from aggregated study-level findings, does not pool standardized effect sizes, and is not an individual-level diagnostic or prognostic biomarker. Translation will require harmonized acquisition and parcellation, normative reference datasets, prospective validation, and integration with diffusion MRI, functional connectivity, inflammatory measures, and clinical trajectories.

## 4 Burnout across functional, transcriptomic, and neurotransmitter scales

Song et al. provide the most explicitly multiscale contribution in the Research Topic. Fifty-one female nurses meeting burnout criteria and 51 matched controls underwent 3 T rs-fMRI. The authors quantified fractional amplitude of low-frequency fluctuations (fALFF) and seed-based functional connectivity, then related the resulting spatial patterns to burnout dimensions, Allen Human Brain Atlas gene-expression data, and PET/SPECT-derived neurotransmitter maps. Nurses with burnout showed reduced precuneus fALFF and reduced precuneus-right dorsolateral prefrontal cortex (DLPFC) connectivity. Lower precuneus fALFF was associated with greater emotional exhaustion and depersonalization and with lower personal accomplishment, while weaker precuneus-DLPFC connectivity was associated with emotional exhaustion.

The circuit-level result implicates impaired interaction between a posterior DMN hub and executive-control cortex, offering a plausible systems account of disrupted self-referential processing, detachment, and reduced top-down regulation in burnout. Spatial transcriptomic analysis associated the fALFF alteration map with gene categories related to membrane depolarization, ion-channel function, synaptic organization, stress signaling, and immune modulation. The same imaging pattern showed spatial correspondence with serotonin, norepinephrine, GABA, glutamate, and endocannabinoid receptor or transporter distributions. These findings add biological depth, but they remain spatial associations with normative postmortem and molecular atlases rather than direct measurements from the participating nurses. Accordingly, they should be regarded as hypothesis-generating molecular priors, not as confirmed molecular mechanisms or immediately actionable treatment targets. Replication in independent, sex-diverse cohorts and longitudinal intervention studies incorporating PET, magnetic resonance spectroscopy, peripheral biomarkers, sleep, and workload measures would substantially strengthen causal and translational inference.

## 5 Alexithymia in multiple sclerosis as a disconnection phenotype

Ayache and Chalah review alexithymia in MS, a construct characterized by difficulty identifying and describing emotions and by externally oriented thinking. Reported prevalence ranges from approximately 10% to 53%, with more recent studies commonly identifying substantial rates across MS, clinically isolated syndrome, and radiologically isolated syndrome. The review links alexithymia to depression, anxiety, fatigue, emotion-regulation difficulties, maladaptive coping, reduced empathy, and, in some studies, altered social cognition or moral judgment.

The neurobiological evidence is still limited but converges on a distributed disconnection account. Structural MRI studies associate higher alexithymia with lower corpus callosum, thalamic, pallidal, deep white-matter, brainstem, cerebellar, and cerebral white-matter volumes. Task-fMRI findings implicate orbital inferior frontal, subgenual anterior cingulate, caudate, amygdala, and frontolimbic connectivity during emotional appraisal and cognitive reappraisal. A small transcranial magnetic stimulation study further reported shorter cortical silent periods in patients with higher alexithymia, consistent with altered cortical inhibitory physiology. These observations suggest that alexithymia in MS may arise from disrupted integration among interoceptive, limbic, prefrontal, and interhemispheric systems. However, the evidence base includes only a small number of structural and functional imaging studies, often with modest samples and cross-sectional designs. A central unresolved question is whether alexithymia primarily reflects MS-related neuropathology, chronic disease-related stress, a pre-existing trait, or an interaction among these factors.

## 6 Cross-cutting synthesis and methodological priorities

Across the four articles, three organizing principles emerge. First, affective dysfunction is network-based. The recurrent involvement of the DMN, prefrontal executive systems, thalamic relay structures, sensorimotor cortex, and callosal pathways is more consistent with disturbed coordination among systems than with isolated regional lesions. Second, affective phenotypes are embodied. The IBS study places depressive symptoms within brain-gut and sensory-interoceptive circuits; the burnout study links self-related processing to occupational stress; the MS review emphasizes interoception and interhemispheric transfer; and the mTBI synthesis situates affective symptoms within distributed structural damage. Third, explanatory depth increases when imaging scales are integrated, but each scale carries distinct inferential limits. The recurrence of midline and prefrontal hubs should not be interpreted as evidence for a universal disease-specific biomarker. These regions have high participation across multiple cognitive and affective systems and may therefore represent common points of vulnerability or compensation. Their transdiagnostic relevance is scientifically valuable, but specificity must be established through direct comparisons, normative modeling, out-of-sample prediction, and tests of whether imaging features explain clinically meaningful variance beyond demographic, behavioral, and symptom measures.

Future affective neuroimaging should prioritize longitudinal and perturbational designs over cross-sectional association alone. Multimodal protocols should combine structural MRI, diffusion imaging, resting state and task fMRI, EEG or MEG, autonomic physiology, and molecular or peripheral biomarkers when mechanistically justified. Dynamic connectivity, effective-connectivity models, structural-functional coupling, and individualized network mapping may help resolve temporal and person-specific heterogeneity. Multi-site harmonization, preregistration, transparent preprocessing, motion control, correction for multiple comparisons, spatial-autocorrelation-aware inference, external validation, and calibrated uncertainty should be treated as prerequisites for biomarker claims. Clinical translation further requires a shift from group-level statistical differences to individual-level decision utility. Candidate signatures should be evaluated for reliability, incremental validity, sensitivity to intervention, fairness across sex, age, ethnicity, disease stage, and socioeconomic context, and robustness across scanners and analytic pipelines. Neuroimaging-guided neuromodulation, including TMS, transcranial electrical stimulation, and neurofeedback, is promising, but target selection should be based on replicated network mechanisms and prospective evidence rather than reverse inference from a single cross-sectional map.

## 7 Conclusion

This Research Topic illustrates the maturation of affective neuroimaging from regional localization toward multiscale network explanation. Liu et al. identify a prefrontal-sensorimotor-thalamic pathway linking depressive symptoms to IBS severity; Mavroudis et al. describe a study-level structural network fingerprint of mTBI centered on DMN-limbic-thalamic systems; Song et al. connect precuneus-DLPFC dysfunction in burnout with spatial transcriptomic and neurotransmitter priors; and Ayache and Chalah synthesize a disconnection-based account of alexithymia in MS. Together, the studies demonstrate that affective disturbances are embedded in interactions among self-referential, executive, interoceptive, limbic, and relay systems. The principal scientific opportunity now is to convert these convergent group-level observations into reproducible mechanistic models and clinically useful individual-level predictions. Achieving this goal will require longitudinal multimodal designs, causal perturbation, rigorous construct validation, harmonized analysis, and explicit separation between neural measurement, statistical association, and subjective experience. Under these conditions, neuroimaging can contribute not merely to descriptive maps of affective dysfunction, but testable accounts of how emotion-related brain systems fail, compensate, and respond to intervention across neurological, psychiatric, somatic, and occupational contexts.

